# Energy efficient reduced graphene oxide additives: Mechanism of effective lubrication and antiwear properties

**DOI:** 10.1038/srep18372

**Published:** 2016-01-04

**Authors:** Bhavana Gupta, N. Kumar, Kalpataru Panda, S. Dash, A. K. Tyagi

**Affiliations:** 1 Indira Gandhi Centre for Atomic Research, Kalpakkam 603102, Tamilnadu, India; 2 Department of Advanced Materials Science, Graduate School of Frontier Sciences, University of Tokyo, 5-1-5, Kashiwanoha, Kashiwa, Chiba 277-8561, Japan

## Abstract

Optimized concentration of reduced graphene oxide (rGO) in the lube is one of the important factors for effective lubrication of solid body contacts. At sufficiently lower concentration, the lubrication is ineffective and friction/wear is dominated by base oil. In contrast, at sufficiently higher concentration, the rGO sheets aggregates in the oil and weak interlayer sliding characteristic of graphene sheets is no more active for providing lubrication. However, at optimized concentration, friction coefficient and wear is remarkably reduced to 70% and 50%, respectively, as compared to neat oil. Traditionally, such lubrication is described by graphene/graphite particle deposited in contact surfaces that provides lower shear strength of boundary tribofilm. In the present investigation, graphene/graphite tribofilm was absent and existing traditional lubrication mechanism for the reduction of friction and wear is ruled out. It is demonstrated that effective lubrication is possible, if rGO is chemically linked with PEG molecules through hydrogen bonding and PEG intercalated graphene sheets provide sufficiently lower shear strength of freely suspended composite tribofilm under the contact pressure. The work revealed that physical deposition and adsorption of the graphene sheets in the metallic contacts is not necessary for the lubrication.

Reduction of friction in mechanical elements is one of the most important factors for improving energy efficiency of machine, enhancing the durability of the components and minimizing the emission of harmful gases in the environment. The automobile and transport industry faces challenges due to the emission of carbon monoxide and other chemically poisonous substances that substantially increase the global warming and risk level for living beings and equally it is hazardous for environment^1^. Layered materials such as graphene^2^,^3^,^4^ graphite^5^,^6^,^7^, hexagonal boron nitride (*h*-BN)^4^, and 2H molybdenum disulphide^8^,^9^ are proven as effective solid lubricants. The unique anisotropic crystal structure of layered materials signifies strong covalent intralayer and weak van der Waals interlayer interactions which are the main reasons for the effective solid state lubrication^2^,^3^. Bahareh Yazdani *et al.* reported enhanced tribological properties of graphene embedded with Al_2_O_3_ composite^10^. In other work, Hyo Jin Kim *et al.* reported synthesis of unoxidized graphene/alumina composite materials having enhanced toughness, strength, and wear-resistance^11^. Unfortunately, the solid phase lubrication of lamellar materials is restricted to the specific application and, therefore the use of these materials in liquid phase lubrication as additives becomes an important issue. Among the above mentioned layered materials, graphene is considered as unique material due to its high chemical inertness, extreme strength, and easy shear capability on its densely packed and atomically smooth surface^2^,^4^. It can exist stable in ultrathin state and dispersion in lubricant makes it a potential material for low friction and wear resistant additives^12^,^13^,^14^. For the effective lubrication, the covalent/non-covalent interaction of graphene with lubricant is required^15^,^16^,^17^. However, such interactions of hydrophobic graphene are challenging in hydrophilic polar lubricant due to difference in cohesive energy. Graphene consists of sp^2^ carbon atoms with delocalized π electrons in 2D network, which is chemically inert and easily agglomerates due to the π*-*π stacking interaction^18^. This becomes one of the main problems dispersing it with polar lubricant. However, higher specific surface area of lamellar materials enhance the ability towards chemical functionalization and better dispersibility in lube medium. The π electrons become localized while creating the oxygen functionlization leading to 2D sheet chemically active^19^. There are several reports which showed functionalized graphene as a compatible lube additive for friction and wear reducing additives^15^,^16^,^17^,^20^. The functionalized graphene provides chemical grafting with lube facilitating weak shear forces acting between the lamellar sheets^16^,^17^,^21^,^22^,^23^. Large amount of graphene concentration in the dispersive medium poorly lubricate the sliding system, generating high friction and wear. Graphene agglomerates and precipitates if the concentration is higher which does not provide the well known mechanism of weak interlayer sliding resistance of graphene sheets. In addition, higher concentration is responsible for shear thickening and inter-sheet collision that may produce high friction and wear^24^. In contrast, at low graphene concentration, the effective lubrication becomes weak due to scarcity of graphene sheets and in this condition lubrication is mainly governed by the lube medium. Therefore, optimized graphene concentration as an additive in the lubricant becomes one of the puzzling issues for effective lubrication. Its mechanism for controlling friction and wear becomes even more puzzling topic. There are few reports which showed concentration dependent friction and wear behavior in graphene/graphene oxide additives in lube medium^15^,^16^,^17^,^20^,^25^. However, lubrication of graphene and concentration dependent lubricity mechanism in the literature is poorly understood. Schluter *et al.* showed that thermally reduced graphene oxide (TRGO) containing lubricant significantly enhanced the friction and wear properties^26^. It is predicted that the enhancement in above properties are attributed to the interaction of amphiphilic additive and functionalized TRGO filler particles on metal oxide surfaces.

In this novel and pristine work, friction and wear of steel-steel contact was proposed to investigate which is lubricated with various concentration of graphene dispersed PEG200 at various loads. The contact pressure was varied to find the generalized experimental proof for concentration dependent lubrication mechanism. Contact surface was analyzed by electron dispersive spectroscopy mapping and local area Raman mapping of wear tracks for describing chemical nature of tribofilm formation. Based on experimental results, the lubrication model in graphene lubricated contacts is proposed.

## Results and Discussion

### Morphology, microstructure and chemical characteristic of rGO

**Figure 1:**
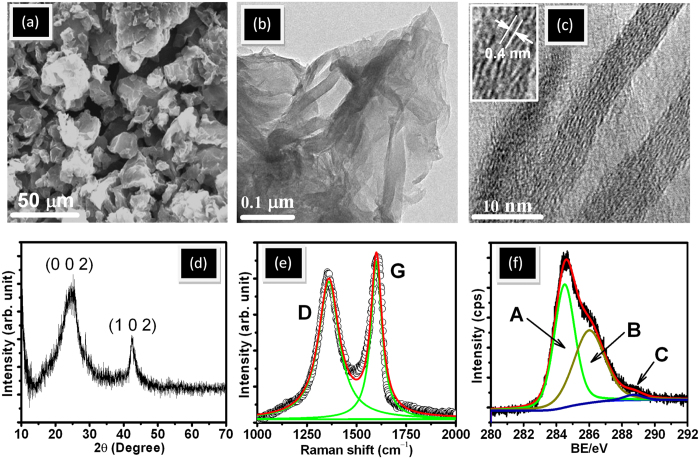
SEM image (a) low resolution TEM of the rGO (b) high resolution TEM of the rGO (c) XRD (d) Raman spectra (e) and C1s of XPS analysis (f).

**Figure 2:**
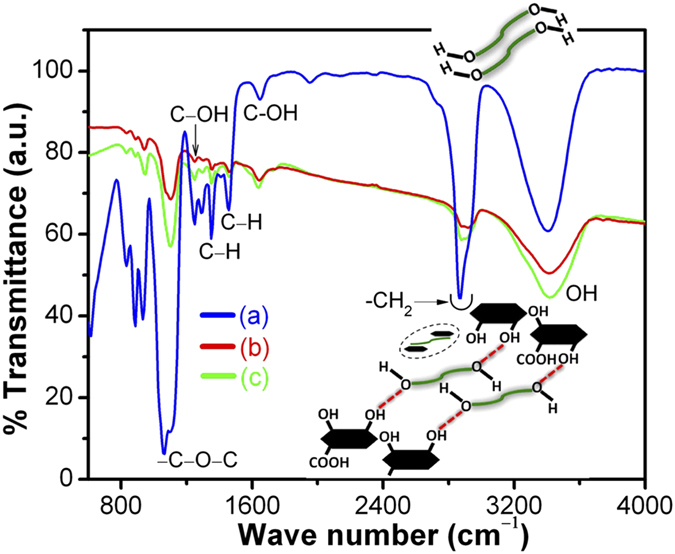
FTIR of (a) PEG200 (b) PEG-rGO, concentration 0.2 mg. mL^−1^ and (c) PEG-rGO, concentration 1.0 mg. mL^−1^.

SEM image clearly shows curly flakes signifying graphene features (Fig. 1a). Further, low resolution TEM shows wrinkles, lateral corrugations and scrolled morphology of rGO, indicating presence of few layers of graphene sheets where strain is localized (Fig. 1b). The high resolution TEM image clearly reveals bunches of crystalline rGO nanosheets with thickness 5–6 nm and number of sheets ranging from 12–15 (Fig. 1c). The lateral dimension of the sheet is significantly larger than the thickness of the sheet. The lattice fringes show interlayer distances of 0.4 nm which belongs to the (002) plane. This is supported by the XRD data showing a diffraction peak at 24.5° 2θ of (002) plane with lattice spacing 0.4 nm (Fig. 1d). The diffraction from (102) plane are observed at 42.45° 2θ and the FWHM of this plane is a measure of lateral dimension of the sheet which is approximately 68 nm. This is well in agreement with high resolution TEM result. In rGO, the D and G bands are observed at 1343 and 1593 cm^−1^, respectively (Fig. 1e). It is known that the D band originates from the edges and it is attributing to defects^27^,^28^. The G band corresponds to the first-order scattering of the E_2g_ mode of sp^2^ domains present in graphite structure. The relative strength of D band as compared to G band depends strongly on the amount of disorder present in the graphite material. In this case, the intensity ratio I(D)/I(G) in rGO is 1.05 signifying defect and disorder in structure due to the oxygen functionalization. To investigate the chemical composition, the XPS of the rGO is carried out (Fig. 1f). The C 1s spectra is de-convoluted into three chemically shifted components designated as A, B and C at binding energies of 284.4, 286 and 288.6 eV, respectively. Component A is non-oxygenious carbon in C–C/C=C configuration, component B is assigned to carbon atoms bonded to the oxygen in hydroxyl (C–OH) or epoxide (C–O–C) and component C shows a small amount of carbonyl (>C=O) and carboxyl groups (COOH or HO–C=O)^29^,^30^,^31^. The sp^2^ fraction in rGO is approximately 70% and remaining carbon atoms is sp^3^ hybridized connected with hydroxyl, epoxy, carbonyl and carboxyl groups. FTIR characterization is unique method for understanding the functionlization of rGO and its interaction with PEG molecules. In PEG, strong band at 2868 and 2932 cm^−1^ corresponds to –CH^2^ (antisymmetric) and –CH^2^ (symmetric) stretching vibration in alkyl chains, respectively, and 1062 cm^−1^ is assigned to vibration of –C–O–C groups (Fig. 2a). Weak signal of alkyl chains are appeared in rGO-PEG dispersed sample, indicating formation of hydrogen bonding between rGO and PEG molecules (Fig. 2b,c). A strong band at 1651 cm^−1^ originates from vibration of C-OH group^32^. These peaks are observed in rGO-PEG dispersed sample and are attributed to coordination between PEG with functionalized rGO through hydrogen bonding. Strong band at 1342 and 1460 cm^−1^ are attributed to vibrations of C–H group and C–OH vibration at 1245 cm^−1^ occurs in PEG molecules. These two bands are also exhibited in rGO-PEG sample and could be a combination frequency of CH + OH group. An absorption band of –C–O–C–at 1062 cm^−1^ is observed in PEG while this peak is also appearing in rGO-PEG indicating cross-linking between PEG and rGO.^33^ Band at 3407 cm^−1^ is due to stretching O–H mode of C–OH group in PEG and these bands are also present in functionalized graphene samples. Therefore, shifting of the band and its widening clearly indicate the presence of hydrogen bonding between PEG and functionalized rGO sample^33^,^34^. The above results are the clear signature of dispersion of rGO in PEG through hydrogen bonding.

### Lubrication properties of rGO additives in PEG

**Figure 3:**
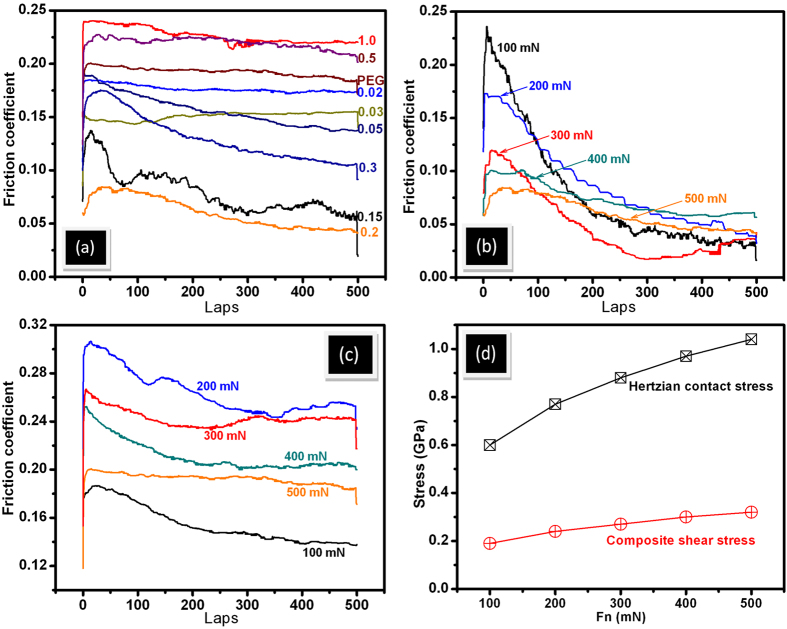
Friction coefficient vs rGO concentration in PEG (a) load dependent friction coefficient of 0.2 mg mL^−1^ of rGO concentration (b) load dependent friction coefficient of PEG200 (c) and load vs Hertzian contact and composite shear stress (d).

**Figure 4:**
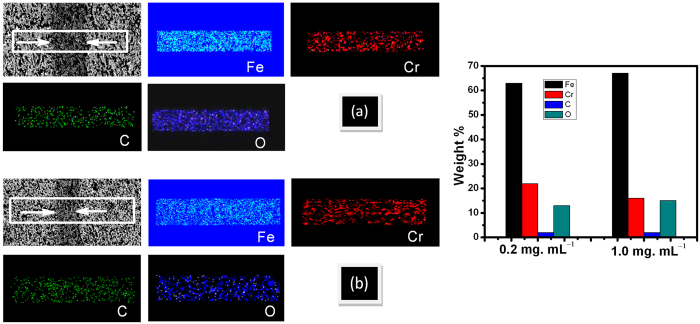
EDS mapping across the wear track and weight % of elements at rGO concentration (a) 0.2 and (b) 1.0 mg mL^−1^, Tribology condition: Load: 500 mN, Linear speed: 0.5 cm/s, Ball: steel (dia. 2 mm).

**Figure 5:**
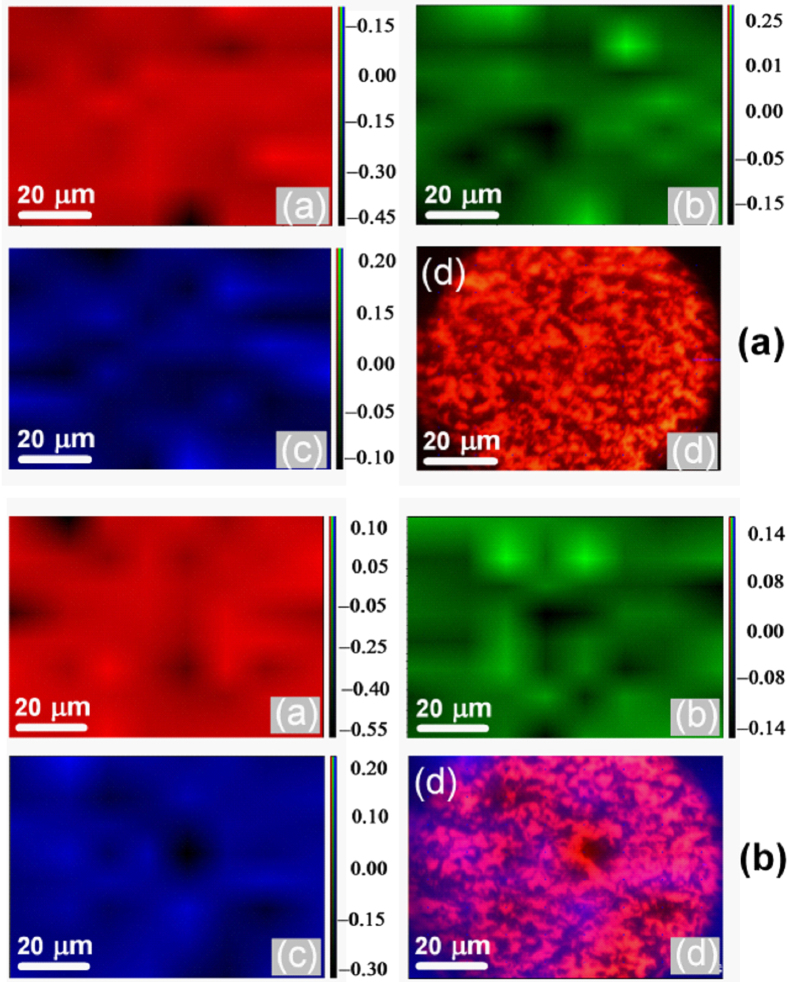
Raman mapping inside the wear track at rGO concentration (a) 0.2 and (b) 1.0 mg mL^−1^. Sublevel both in figure (**a**) and (**b**) describes: (**a**) metal oxide (**b**) D band (**c**) G band and (**d**) superimposed mapping pattern of D and G band, the scale in image show intensity. Tribology condition: Load: 500 mN, Linear speed: 0.5 cm/s, Ball: steel (dia. 2 mm).

Friction coefficient of dry steel-steel contact is approximately 0.6 (Figure S1) and this value is reduced to 0.2 in neat PEG steel-steel lubricated condition at 500 mN load (Fig. 3a). This value decreased up to 7% using minute concentration 0.02 mg mL^−1^ of rGO in PEG. The reduction is significant where friction coefficient decreased to 0.06 i.e. 78% less at 0.2 mg mL^−1^ rGO concentration. The concentration dependent trend of friction values is shown in Figure S2. In addition, wear scar is reduced to almost 50% in 0.2 mg mL^−1^ rGO concentration as compared to neat PEG (Figure S3). Further, increase in concentration, the friction coefficient increases and becomes 0.22 at 1.0 mg mL^−1^ of rGO. Wear depth also increases in the same trend as friction coefficient (Figure S2, S3). It is worth to mention that optimized concentration of rGO is not a universal value. It largely depends on physical and chemical properties of the solid-body contact and contact stress. In early report, lowest value of friction coefficient was achieved in 0.03 mg. mL^−1^ concentration while sliding between 100Cr6 steel ball and 316LN stainless steel disc^15^,^17^. However, concentration dependent trend of friction and wear is observed almost for any conventional additive in the oil or polymer matrix. It is usually determined by change in viscosity at low concentrations and degradation of lubricity at high ones. Here, generally, the viscosity (dynamic and kinematic) of rGO dispersed in PEG200 does not much change with the concentration of rGO additives (Figure S4). Neat PEG (shown by arrow in Figure S4) and PEG with rGO concentration 0.02 mg. mL^−1^ do not show any changes in viscosity. Further, with increase in concentration up to 0.3 mg. mL^−1^, the slight increase in viscosity is shown and this is related to good dispersibility of rGO additives (dispersion stability will be shown in later section). However, at higher concentration, the viscosity value is almost saturated, indicating no more chemical dispersion of rGO additives is further possible in PEG. Here, the result indicates that the role of viscosity in friction and wear is not a dominant factor. This is well corroborated by contact angle measurement which also does not change with concentration and slightly varies in the range 53° to 56° (Figure S5). This indicates that balance of rGO polarity is retained in the respect of PEG where concentration does not play a major role. Analogically, the tribologically effective concentration 0.2 mg mL^−1^ of rGO was selected to analyze load dependent friction stability (Fig. 3b). The trend of friction coefficient vs sliding cycles was more or less similar at all the loading condition while with increase in load, the initial value of friction coefficient decreases. This clearly describes decrease in standard deviation of friction coefficient with increasing load (Figure S6a). This is possibly an indication of (a) alignment of graphene in the contact region and (b) shear induced plastic deformation of surface asperities, leading to contact smoothening. In microscopic scale, a lamellar graphite-like structure is formed with in-plane sp^2^ bonding and weak interlayer coupling. Thus, the decrease in friction is a result of diminishing and ultimately vanishing interlayer bonds. In this condition, the selforganizing response to shear action and formation of highly ordered and lamellar structure are observed^35^. The virgin contact surfaces represent rougher asperities (values are given in the characterization section) that plastically deformed during the initial sliding cycles and in this regime, the friction energy is dominated by plastic deformation. At high loads, the rough asperities suppose to break down, suppressing the extent of plastic deformation, attributing to decrease in friction value (Fig. 3b). In the similar experiment, the value of friction coefficient is high in PEG lubricated condition but the trend is similar to the 0.2 mg mL^−1^ rGO lubricated condition (Fig. 3c). However, at 100 mN load, the friction of PEG lubricated system is lower. Here also, the standard deviation of friction coefficient becomes less at high loads (Figure S6b). It is shown that with increase in load, Hertzian and composite shear stress are increased that help to (a) align the graphene sheets and (b) suppress the plastic deformation of surface asperities (Fig. 3d). The possible reason of such alignment will be discussed below. Graphene sheets may lead to the formation of a boundary tribofilm in the contact and therefore, the lubrication of PEG effectively works. In addition, better alignment of the linear PEG molecules cannot be ignored at high pressure that provides low shear resistance of the sliding plane and therefore, load carrying capacity was improved with decreased wear depth (Figure S7). This is effective both in base oil and rGO dispersed PEG lubricated system which clearly indicates that this property is inherently present in PEG molecules. In order to explain friction and wear behavior, two wear tracks formed in 0.2 and 1.0 mg mL^−1^ concentration was used for analysis. For better understanding, these wear tracks will be designated as W1 and W2 respectively. Selection of these tracks is logical because 0.2 mg mL^−1^ rGO showed lowest value of friction coefficient (0.02) while 1.0 mg mL^−1^ rGO has shown highest friction value (0.22). In order to confirm the deposition on the wear track, the EDS analysis is performed across the W1 and W2 wear tracks. The motivation behind this analysis was to observe the distribution of carbon concentration in the wear track along with surface region of steel. It is shown that across the steel surface to wear track, there is no difference in carbon concentration (Fig. 4). The average elemental weight % of the mapped region in both the concentration shows negligible amount of C that mostly belong to residual amount of carbon present in T42 HSS steel disc. Signal of carbon is same in selected region, indicating absence of any carbon deposition in the track. The signal of other elements such as Fe, Cr and O is also having uniform distribution in the selected region. For quantitative chemical analysis, the extensive Raman mapping inside the wear track is carried out (Fig. 5). Selected frequency range was used to detect the possible phases like metal-oxide at (a) 600–700 cm^−1^ (red), D and G band of carbon (Fig. 5b) and (Fig. 5c) at 1300–1400 cm^−1^ (green) and 1500–1600 (blue), respectively. Superimposed mapping pattern of these phases are represented in (Fig. 5d) of both the figures. In both the wear tracks, D and G bands along with few very weak signal of metal oxide are observed. The individual spectra of the typical features mostly do not show above mentioned characteristic of phases (Figure S8a,b). Such a broad and weak signal of D and G band is signature of elemental carbon inherently present in steel sample. This indicates absence of any physisorbed/chemisorbed graphene/graphite substances in the wear track.

### Lubrication mechanism

**Figure 6:**
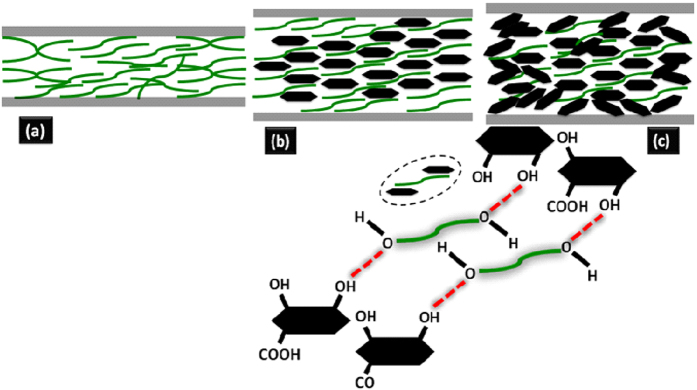
Lubrication mechanism in (a) PEG steel-steel contact (b) 0.2 mg mL^−1^ rGO-PEG lubricated contact and (c) 1.0 mg mL^−1^ rGO-PEG lubricated contact.

**Figure 7:**
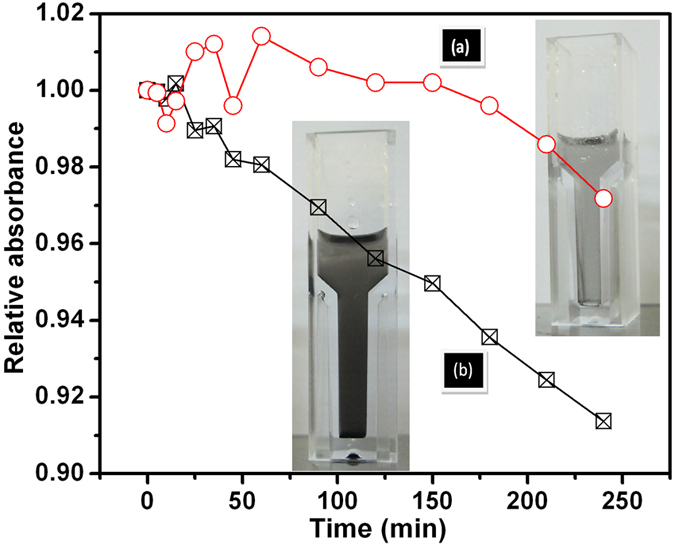
Suspension stability of the lubricating oils with rGO concentration (a) 0.2 and (b) 1.0 mg.mL^−1^ as determined by ultraviolet–visible light (UV– VIS) spectrophotometry.

**Figure 8:**
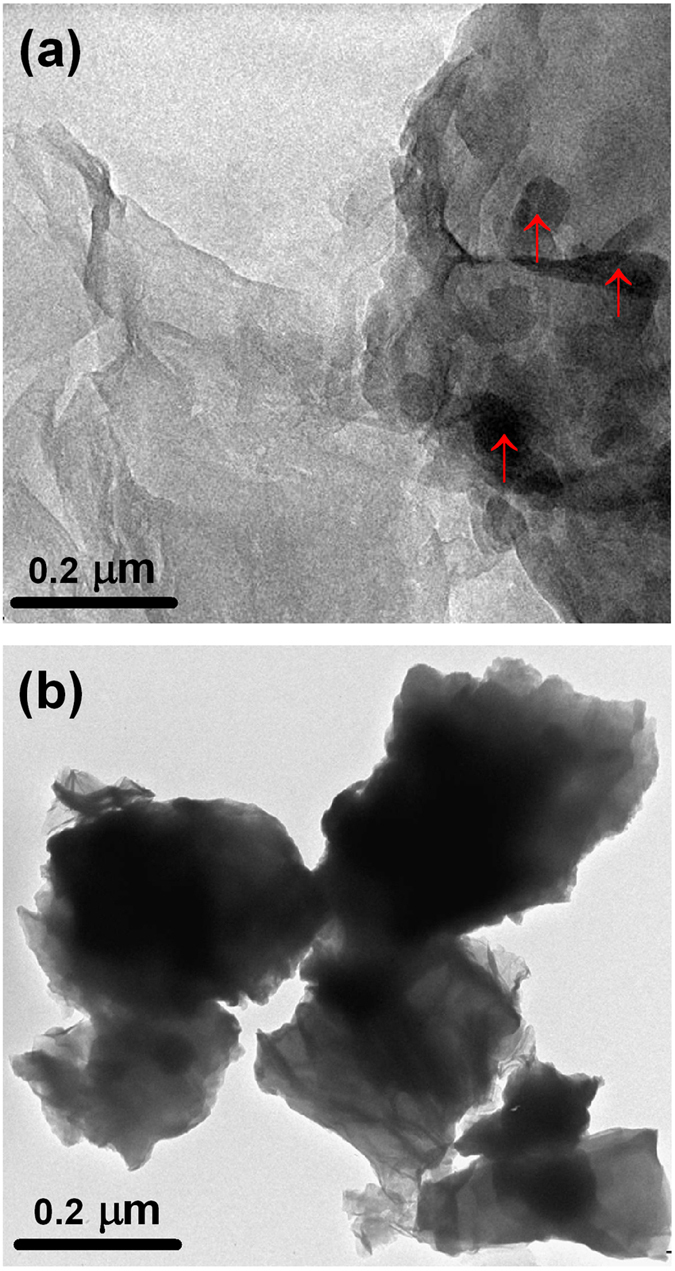
Morphology and dispersion stability of the lubricating oils with rGO concentration (a) 0.2 and (b) 1.0 mg.mL^−1^ analyzed by TEM.

Several reports showed that the adsorbed graphene/graphite tribofilm formation in the contact plays the key role for the reduction in friction and wear^15^,^16^,^21^,^25^,^36^,^37^. Such reduction was realizable only at optimized concentration of graphene dispersed in the lube^15^,^16^,^17^,^25^. Also, adsorbed graphite tribofilm was observed at high concentration but it was not participated in the lubrication process^17^. It is argued that graphene is agglomerated when concentration is high and due to this the weak shear strength of the interlayer sliding plane disrupts. The question arises about the physical reason behind the deposition of graphene in the metallic contact. Primarily, it is useful to take a contact stress in account that possibly helps for deposition. It was shown that rGO dispersed in PEG was found to well deposited in the steel contact in microtribometric test^15^,^17^, but here it is unable to deposit in nanotribometric contact where contact stress is higher. It is worth to mention that ball diameter was 6 mm in microtribometric experiment^15^,^17^. In this case, the calculated Hertz stress and shear stress is 0.65 and 0.2 GPa, respectively and these values are well below to stress observed in nanotribometric contact condition where diameter of the ball is 2 mm and applied force 500 mN (Fig. 3d). Therefore, the possibility to deposit the graphene film is higher in high contact stress condition i.e. nanotribometric contact. At high stress, shear ability of graphene sheets enhances due to repulsive Coulomb interaction. Interfacial repulsions can also originate from *π* orbital interaction between adjacent sheets in graphite, which has been reported to possess superlow friction at large contact stress^38^,^39^,^40^. In addition to contact pressure which is invalid, the deposition of graphene/graphite should be related to plastic deformation of the steel contact which is high in microtribometric test condition. This is shown in the work reported by Gupta *et al.*^17^. In this report, the 100Cr6 steel ball, diameter 6 mm and hardness 2.3 GPa was used against 316 LN steel. Plastic deformation releases the high energy and it is useful for the physisorption and chemisorptions of graphene/graphite dispersed PEG molecules. Based on the experimental fact, it is obvious that graphene/graphite deposition is mediated by plastic deformation of the metallic contact^17^. Mild deformation is observed where scratches, grooves and adhesive failure are insignificant in the wear track of nanotribometric contact (Figure S9). However, linear scratches and grooves are clearly visible in neat PEG contact and at higher concentration rGO-PEG contact. In neat PEG lubrication, boundary film squeezes and does not have sufficient ability to bear the load (Fig. 6(a)). Literature reports mostly argue that lubrication of dispersed graphene/graphite is effective if these substances are deposited in the contact region which easily shears under the action of contact stress^15^,^16^,^21^,^25^,^36^,^37^. However, in the present work, extensive analysis of wear track does not show deposited graphene/graphite substances. In this condition, lubrication can be defined by proposing other relevant model. It is clearly shown in FTIR studies, that rGO is linked with PEG molecules through hydrogen bonding (Fig. 2). Under the contact pressure, the graphene could be aligned along the linear direction of PEG molecules without disturbing the hydrogen bonding linkages which is schematically shown in Fig. 6b. In solid graphene, the lubrication is basically mediated by low shear strength which is acting between the graphene sheets. The structure and morphology of grapehene is stable and shear mechanism is active when rGO is well dispersed in the oil. Undispersed graphene made aggregated particles and shearability is disrupted. The dispersion stability is high in 0.2 mg.mL^−1^ rGO concentrations, where absorbance decreases marginally with time (Fig. 7a). Digital images were obtained after 240 minutes that clearly shows well dispersed rGO of concentration 0.2 mg.mL^−1^ in the oil. For a comparison, the shear rate vs shear stress relationship of neat PEG and PEG with 0.2 mg.mL^−1^ graphene concentration shows a characteristic of definite Newtonian liquid-like behavior (Figure S10). Comparatively, graphene dispersed PEG sample clearly shows less shear stress which could be related to less shear strength of graphene lamella interacting to the PEG molecules. Under the contact pressure, the alignment stability of graphene sheets is mostly controlled by PEG molecules and it may forms stable composite boundary tribofilm of PEG intercalated graphene sheets. Mainly at high contact pressure, the shear mobility of the graphene sheets in lube becomes effective, providing efficient lubrication and load bearing capacity. At higher rGO concentration, the lubrication is ineffective, increasing the friction and wear adversely, as it is mechanistically presented in Fig. 6c. This could be explained by defining two possible reasons. Firstly, at high concentration, undispersed particles are agglomerated and then losing its alignment. The poor absorbance properties of 1.0 mg mL^−1^ rGO indicate time dependent agglomeration of the rGO in oil (Fig. 7b). This is a signature of weak dispersion stability and therefore, viscosity does not change much. The TEM analysis shows that at higher concentration of 1.0 mg.mL^−1^ graphene, the whole region is heavily loaded by graphene flakes and it becomes darker, indicating agglomeration (Fig. 8b). This may interrupt shear mobility between weak interlayer graphene sheets. In this condition, PEG molecules do not participate to form a composite boundary tribofilm lubricant. Secondly, the agglomerated randomly oriented graphene particles physically form the obstacles between the sliding surfaces while intercepting the interfacial sliding. It is worth to mention that composite boundary tribofilm is freely suspended in the contact and do not deposit in metallic contact. However, at concentration 0.2 mg.mL^−1^, the TEM results show transparent, wrinkled and curly feature which is typical morphology of graphene sheets. Only small region is heavily loaded by graphene as indicated by arrow Figure 8a. This clearly indicates that effective dispersion of graphene need optimized concentration in PEG based lube.

## Conclusions

Concentration of rGO additives in the lube is an important aspect for controlling the friction and wear of the lubricated solid contacts. At lower and higher concentration, the lubrication is ineffective, producing higher friction and wear. However, in optimized concentration 0.2 mg.mL^−1^ of rGO in lube medium showed extremely lower value of friction coefficient 0.06 and wear reduced up to 50% as compared to neat PEG lubricated steel-steel contact. Moreover, at high contact pressure, the energy efficiency of optimized rGO additives is improved. This is related to effective shearing of graphene dispersed in PEG through formation of low shear strength boundary film. It is shown that boundary film in the form of graphene/graphite was not deposited in the contact region. Therefore, an effective lubrication properties are described by freely suspended boundary tribofilm formation of PEG intercalated composite graphene sheets. The graphene sheets are linked with PEG molecules through hydrogen bonding and under the contact stress, low shear strength of interlayer sheets easily slides, providing effective lubrication. The graphene sheets along the PEG molecules become superior at high contact pressure, reducing the friction significantly and more importantly deformation of the wear tracks become negligible.

## Method

### Synthesis of rGO

Synthesis of GO is based on Hummer’s methods followed by its reduction by hydrazine treatment. For the GO synthesis, 3.0 g of graphite powder dispersed in 150 ml of 1M H_2_SO_4_ at room temperature. Subsequently, 8.0 g of KMnO_4_ was added in the reaction mixture under room temperature with constant stirring. After 2 hrs of stirring; 90 ml distilled water was slowly added in the reaction mixture followed by heating at 90 °C for 12 hrs. After 12 hrs of heating, 30% H_2_O_2_ was added in the reaction mixture and allowed for further 2 hrs of stirring. After completion of reaction solid, brownish black precipitated was filtered and washed with 100 ml of 30% HCL and 100 ml of ethanol. Finally, precipitates were dried under vacuum at 60 °C of temperature. For reduction of GO to rGO, 100 mg of GO was mixed in 30 ml of water and treated with 3 ml hydrazine under efflux condition at 95 °C for 24 hrs. After completion of reaction, reaction mixture was filtered, washed and dried similar to that of GO mentioned above.

### Characterizations and analysis

Morphology of rGO was analyzed by scanning electron microscope (SEM) whereas wear track elemental mapping was carried out by energy dispersive X-ray (EDX). High resolution morphology and microstructure of rGO was analyzed by transmission electron microscope (TEM) working in low resolution (LR) and high resolution (HR) mode. Crystallographic details and chemical composition were studied by X-ray diffraction (XRD) using Cu Kα radiation (λ = 0.15418 nm) and X-ray photoelectron spectroscopy (XPS- ULVAC-Phi ESCA1800) coupled monochromatic Mg Kα source (400 W, 1253.6 eV), respectively. Chemical structure of rGO and wear track was mapped locally by micro-Raman spectrometer (Horiba 800) operating in laser wavelength of 514.5 nm. FTIR spectrometer (Bruker Optics), operating in transmission mode with a spectral resolution 4 cm^−1^ was used for the analysis of functional groups in rGO. For this analysis, rGO was dispersed in PEG200 followed by ultrasonication and processed in dry form. The rGO dispersed PEG200 was used as synthetic lube base oil for tribology evaluation. Neat PEG itself is considered useful for lubricating additives^41^,^42^,^43^. Before the tribology test, the PEG functionalized rGO powder sample was thoroughly dispersed in the PEG medium by ultrasonication process for 1 hour and their dispersion stability was monitored for 4 hours. It was carried out at room temperature and ultrasonic frequency of 25 KHz. The viscosity measurement was carried out by Anton Paar rolling ball viscometer (Lovis 2000 M/ME). Rolling time of ball in the viscous liquid is estimated in accordance to Hoeppler’s falling ball principle at room temperature. In each measurement, the 2 ml volume of sample was used. The test was repeated several times and average value is reported. Contact angle measurement was carried out by contact angle meter equipped with CCD camera (Holmarc, HO-IAD-CAM-01, and India). Volume of the water droplet used for contact angle measurement was ∼l μL. The measurement was performed at ambient conditions and room temperature. Standard deviations in contact angle measurements were typically ± 1°. The images obtained were analyzed to measure the contact by using the Image J software. The supernatant fluid decanted at specified time intervals to evaluate absorbance using a UV–VIS spectrophotometer. Based on the Lambert–Beer law, the absorbance is proportional to concentration. Therefore, the dispersion stability of rGO in base oil was evaluated by assessing the absorbance. The tribo-evaluation of dispersed graphene was carried out by measuring friction and wear using a ball-on-disc nanotribometer (NTR^2^, CSM Instrument, Switzerland). A 100Cr6 spherical steel ball with diameter 2 mm and hardness 2.3 GPa was used as a sliding counterbody against the static lubricated high-speed steel (grade T42) disc. This is a wear resistance and high compressive strength material with hardness value 0.7 GPa. This material is useful for machine and industrial application. The chemical composition of 100Cr6 steel ball and T42 grade steel is given in the Table 1. The friction coefficient was determined by a linear variable differential transformer (LVDT) sensor, measuring the deflection in the cantilever spring. The experiments were carried out with change in rGO concentration in PEG medium. The normal force was varied 50 mN to 500 mN, linear sliding speed 0.5 cm/s, wear track radius of curvature 1 mm. In each tribo-test, 5 ml ultrasonicated rGO was dispersed in PEG lubricant and it was thoroughly lubricated the sliding interfaces. Rheological measurements were carried out at 25 °C on MCR301 using a cone and plate geometry of gap 0.052 mm and diameter 25 mm.

## Additional Information

**How to cite this article**: Gupta, B. *et al.* Energy efficient reduced graphene oxide additives: Mechanism of effective lubrication and antiwear properties. *Sci. Rep.* **6**, 18372; doi: 10.1038/srep18372 (2016).

## Supplementary Material

Supplementary Information

## Figures and Tables

**Table 1:** Chemical composition of 100Cr6 steel ball counterbody and T42 grade steel disc used for friction measurement.

(%)	C	Si	Co	Mn	V	W	Cr	Mo	Al	Cu	Fe
100Cr6	0.98	0.2	—	0.32		0.015	1.43	0.1	0.05	0.3	(Balance)
T42 HSS	1.28	0.05	10	0.1	3.1	9.2	4	3.3	—	—	(Balance)
