# Supplementary material for: Energy efficient reduced graphene oxide additives: Mechanism of effective lubrication and antiwear properties
Source: Sci Rep. 2016 Jan 4;6:18372. doi: 10.1038/srep18372 (PMC4698732; doi:10.1038/srep18372)
Supplement: Supplementary Information [file srep18372-s1.pdf]

Supporting Information:

Energy efficient reduced graphene oxide additives: Mechanism of effective lubrication and antiwear properties

Bhavana Gupta[a], N. Kumar[a*], Kalpataru Panda[b], S. Dash[a], A.K. Tyagi[a]

[a]Indira Gandhi Centre for Atomic Research, Kalpakkam 603102, Tamilnadu, India

[b]Graduate School of Engineering, Osaka University, Osaka, Japan

Correspondence and requests for materials should be addressed to N.K. (niranjan@igcar.gov.in, phystribology@gmail.com,Tel.: +91 44 27480500 (ext. 22537), Fax: +914427480081)

Table of Content

Figure S1: Friction coefficient of steel-steel dry contact condition. Load: 500 mN, linear sliding speed: 0.5 cm/s, radius of curvature of wear track: 1 mm, Ball: 100Cr6 steel (2 mm diameter).

Figure S2: Concentrations of rGO vs average friction coefficient. Load: 500 mN, linear sliding speed: 0.5 cm/s, radius of curvature of wear track: 1 mm, Ball: 100Cr6 steel (2 mm diameter).

Figure S3: Concentrations of rGO vs wear depth. Load: 500 mN, inear sliding speed: 0.5 cm/s, radius of curvature of wear track: 1 mm, Ball: 100Cr6 steel (2 mm diameter).

Figure S4: The rGO concentration dependent dynamic and kinematic viscosity of PEG

Figure S5: Contact angle of (a) neat PEG and with various rGO concentration (b) 0.02 (c) 0.05 (d) 0.15 (e) 0.2 (e) 0.3 (g) 0.5 and (h) 1.0 mg. mL^{-1} in PEG.

Figure S6: Standard deviation of friction coefficient vs. normal load in (a) 0.2 mg. mL^{-1}rGO dispersed in PEG200 (b) neat PEG200 lubricated steel-steel contact. This plot is given on the basis of results presented in Figure 3b-c, respectively.

Figure S7: Load dependent wear depth in (a) neat PEG200 lubricated steel-steel contact and (b) 0.2 mg mL^{-1} rGO dispersed in PEG200. Linear sliding speed: 0.5 cm/s, radius of curvature of wear track: 1 mm, Ball: 100Cr6 steel (2 mm diameter).

Figure S8: Raman mapping inside the wear track at rGO concentration(a) 0.2 and (b) 1.0 mg.mL^{-1}, (a-g) represents typical feature of data obtained from the random position of the wear tracks. These data corresponds to the Raman mapping produced in figure 5.

Figure S9: Optical image of wear track (a) PEG lubricated contact (b) 0.2 mg. mL^{-}rGO PEG lubricated contact and (c) 1.0 mg. mL^{-1} rGO PEG lubricated contact.

Figure S10: Shear stress vs. shear rate in (a) pure PEG and (b) PEG with 0.2 mg. mL^{-1} graphene concentration

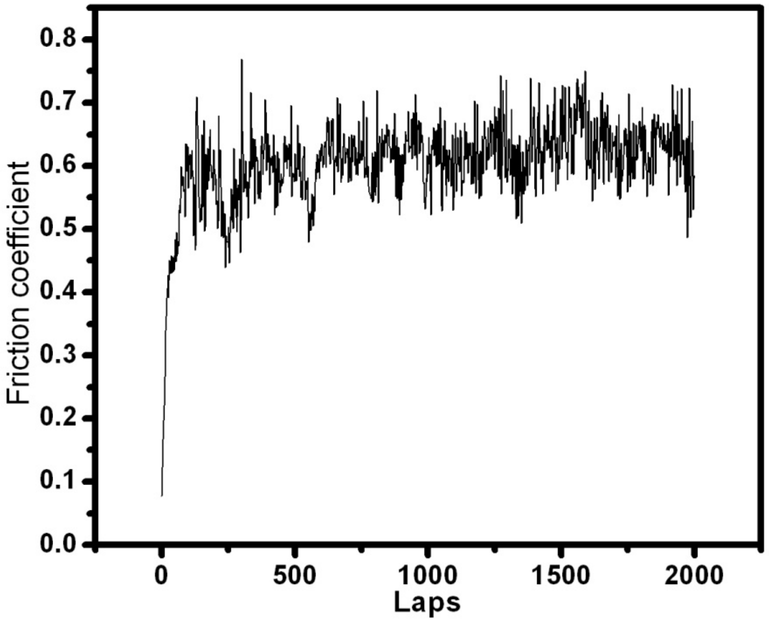

Figure S1. Friction coefficient of steel-steel dry contact condition. Load: 500 mN, linear sliding speed: 0.5 cm/s, radius of curvature of wear track: 1 mm, Ball: 100Cr6 steel (2 mm diameter).

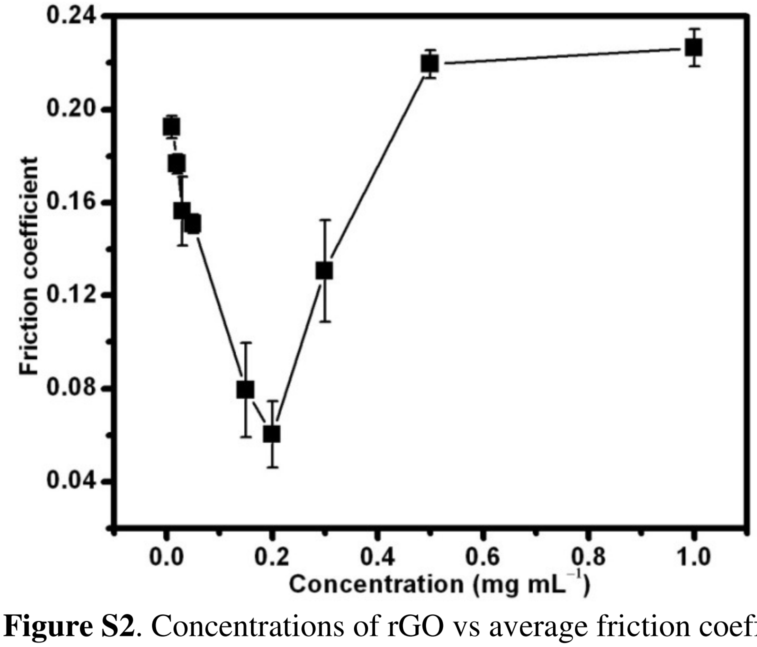

Figure S2. Concentrations of rGO vs average friction coefficient. Load: 500 mN, linear sliding speed: 0.5 cm/s, radius of curvature of wear track: 1 mm, Ball: 100Cr6 steel (2 mm diameter).

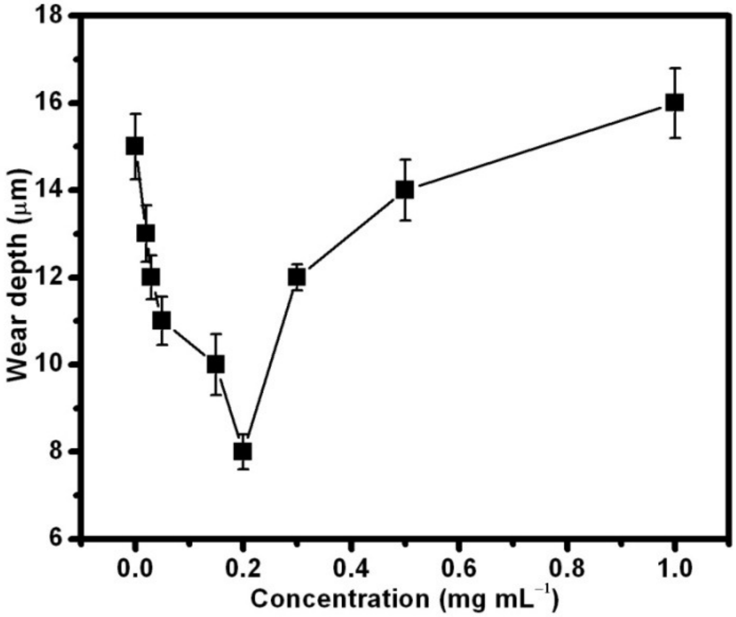

Figure S3. Concentrations of rGO vs wear depth. Load: 500 mN, inear sliding speed: 0.5 cm/s, radius of curvature of wear track: 1 mm, Ball: 100Cr6 steel (2 mm diameter).

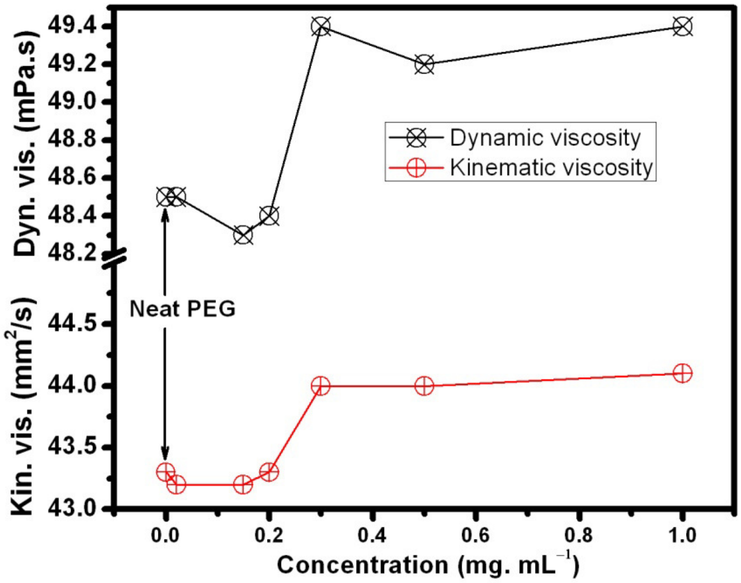

Figure S4. The rGO concentration dependent dynamic and kinematic viscosity of PEG

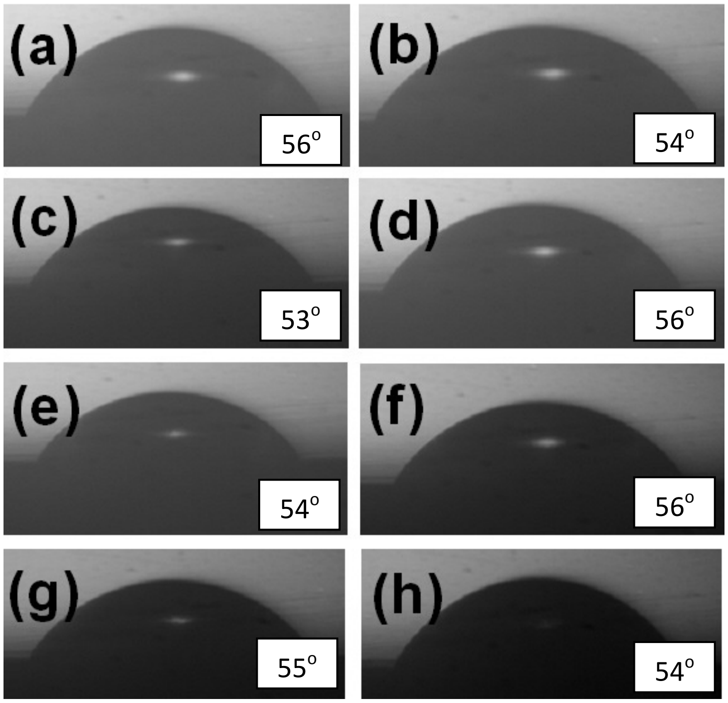

Figure S5. Contact angle of (a) neat PEG and with various rGO concentration (b) 0.02 (c) 0.05 (d) 0.15 (e) 0.2 (e) 0.3 (g) 0.5 and (h) 1.0 mg. mL^{-1} in PEG.

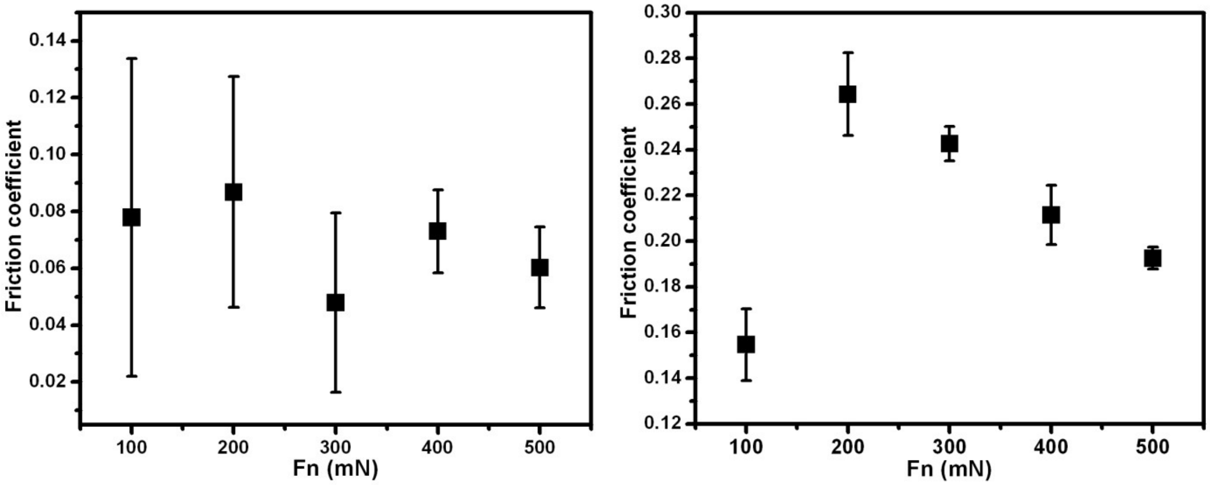

Figure S6. Standard deviation of friction coefficient vs. normal load in (a) 0.2 mg. mL^{-1}rGO dispersed in PEG200 (b) neat PEG200 lubricated steel-steel contact. This plot is given on the basis of results presented in Figure 3b-c, respectively.

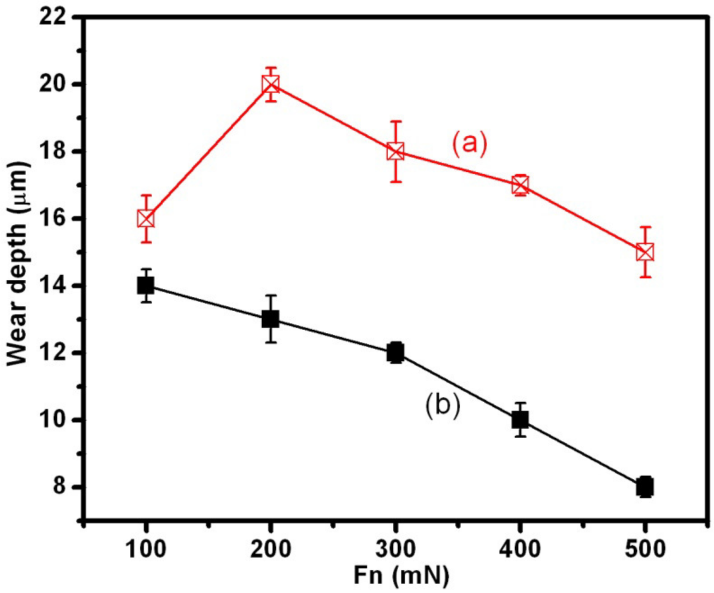

Figure S7. Load dependent wear depth in (a) neat PEG200 lubricated steel-steel contact and (b) 0.2 mg mL^{-1} rGO dispersed in PEG200. Linear sliding speed: 0.5 cm/s, radius of curvature of wear track: 1 mm, Ball: 100Cr6 steel (2 mm diameter).

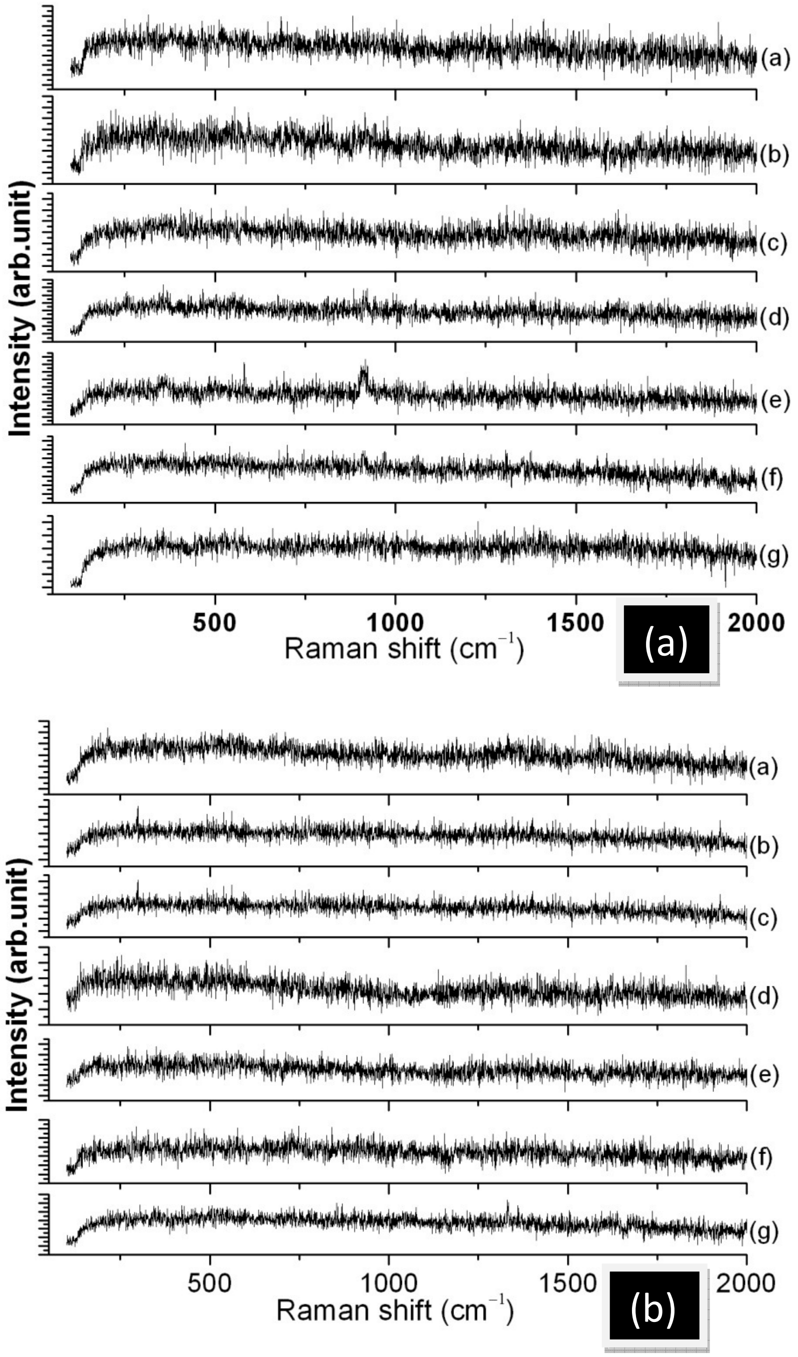

Figure S8. Raman mapping inside the wear track at rGO concentration(a) 0.2 and (b) 1.0 mg.mL^{-1}, (a-g) represents typical feature of data obtained from the random position of the wear tracks. These data corresponds to the Raman mapping produced in figure 5.

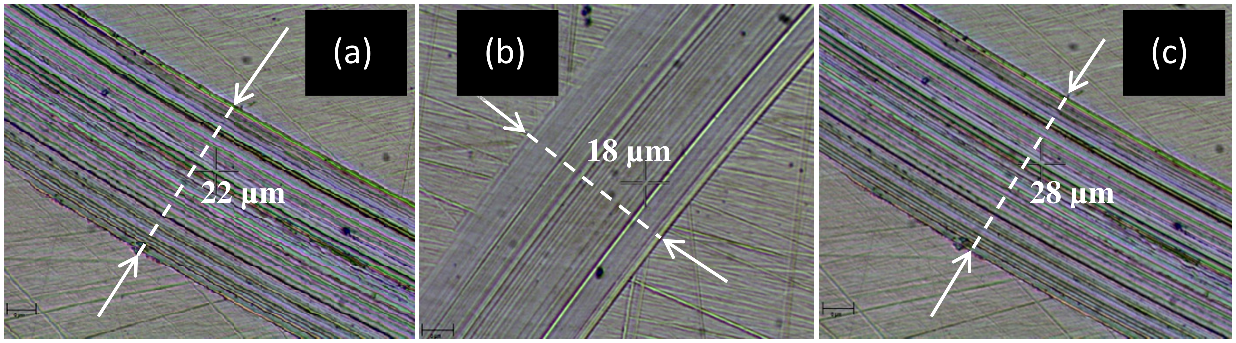

Figure S9. Optical image of wear track (a) PEG lubricated contact (b) 0.2 mg. mL⁻rGO PEG lubricated contact and (c) 1.0 mg. mL^{-1} rGO PEG lubricated contact.

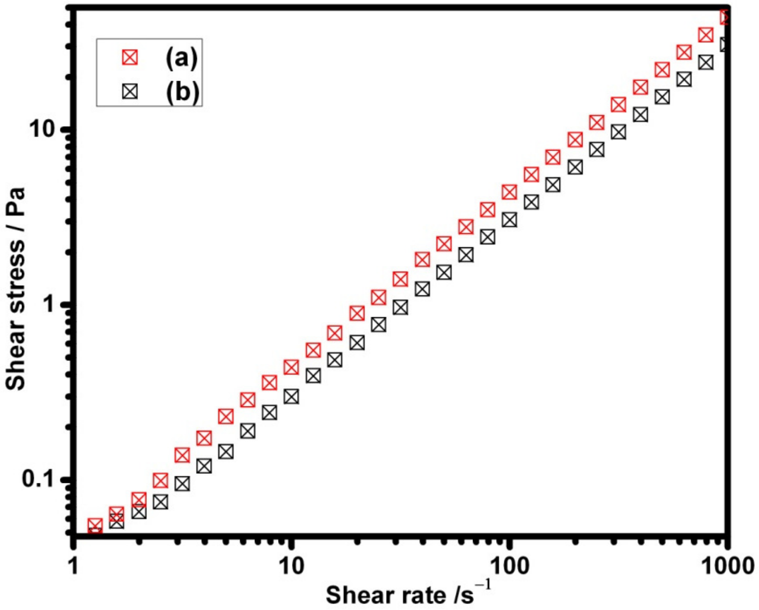

Figure S10. Shear stress vs. shear rate in (a) pure PEG and (b) PEG with 0.2 mg. mL^{-1} graphene concentration
